# Study of sexual behaviours with different types of estrus synchronization protocols in Boer goats

**DOI:** 10.1590/1984-3143-AR2020-0038

**Published:** 2021-08-25

**Authors:** Suraya Mohamad Salleh, Abdul Mu’in Hassan Basri, Halimatun Yaakub

**Affiliations:** 1 Department of Animal Science, Faculty of Agriculture, Universiti Putra Malaysia, 43400 Serdang, Selangor, Malaysia

**Keywords:** estrus synchronization, progesterone, luteinizing hormone, Controlled Internal Drug Release (CIDR), goats

## Abstract

There is still a lack of information on estrus synchronization in goats. Understanding the estrus synchronization protocols and the subsequent effects is important to improve the efficiency of assisted reproductive technologies (ARTs) and subsequently would improve the breeding procedures. This study will help in determining the most suitable estrus synchronization protocol and understand better the effect on the sexual behaviour and hormonal effects in goats. A total of 127 Boer does were used and divided into three groups with different duration of CIDR insertion intravaginally either for 14 (two groups) or 9 days (one group). Approximately 0.5 ml Estrumate^®^ (PG) was administered intramuscularly to all groups at CIDR removal, and only groups PMSG14 and PMSG9 were administered with 200IU of Pregnant Mare Serum Gonadotropin (PMSG) intramuscularly. Estrus signs were observed at 4 h intervals and blood samples were collected for progesterone and luteinizing hormone determination. The percentage of does in estrus within 24 to 72 h post CIDR removal was significantly higher (P<0.05) in groups with PMSG compared to the group without the PMSG. The numbers of does display estrus signs within 24 to 28 h post CIDR removal were significantly higher (P<0.05) in group shorter period (9 days) compared to groups with 14 days CIDR. The P4 concentrations at 24 hours post CIDR removals and LH concentration was not significantly different (P>0.05) in all groups. The time of the LH peak in the group without the PMSG was significantly delayed (P<0.05) when compared to group 9 days CIDR and administered with PMSG. It is recommended to use the treatment for 9 days CIDR since the estrous cycle can be shortened.

## Introduction

Goat meat production is among the important meat besides beef and mutton. Breeds such as Boer, Savannah, and Red Kalahari can adapt to different environmental conditions and grow rapidly with excellent carcass quality (Lu, 2001; Rahman et al., 2008b; Hifzan et al., 2015; Maynard, 2015). A good breeding program needs to be developed to maintain and continuously produce these breeds efficiently.

Assisted Reproductive Technologies (ARTs) have been used in reproductive and breeding protocols to maximize the improvement and the utilization of these breeds (Rahman et al., 2008b). The most common technologies being used are artificial insemination (AI), superovulation, and embryo transfer. Estrus synchronization is an important prerequisite to facilitate the use of these biotechnology techniques efficiently (Martinez et al., 2002). Estrus synchronization in goats has not much attention since early 2000. Hence, related studies are needed to update and ensure the effectiveness of certain protocols for more efficient livestock production specifically in goats.

Many types of research related to estrus synchronization have been conducted in cattle and sheep to determine the best protocol for estrus synchronization. Rahman et al. (2008a) reviewed several estrus synchronization protocols that have been used since 1986. However, the information on estrus synchronization in goats is limited especially in tropical countries. Hence, the present study will contribute to the understanding of different estrus synchronization protocols on estrus behaviour and hormonal profiles. Estrus synchronization is a very important protocol in animal breeding purposes generally. Most importantly, it helps farmers to breed their animals at the same time as well as helps researchers to control and conduct a test at the same time to minimize environmental effects.

The usage of controlled internal drug release (CIDR) along with prostaglandin (PG) to synchronize estrus is a common practice in estrus synchronization in ruminants. Oliveira et al. (2001) and Zeleke et al. (2005) reported that the usage of CIDR was more effective when used along with PG and pregnant mare serum gonadotrophin (PMSG). This resulted in a 100% fertility or success rate when natural mating was used. Oliveira et al. (2001) and Salleh et al. (2014) showed that the addition of PMSG significantly increased the number of animals on estrus. In tropical countries especially, there is still a lack of information on the effects of estrus synchronization using CIDR in goats. Understanding this would increase the efficiency of goat production, by reducing the problem of estrus detection, labour cost, and increase the uniformity of the kids (Oliveira et al., 2011).

The present study was conducted to find the best protocol of estrus synchronization and understand better the differences between different estrus synchronization protocols on the hormones and estrus behaviour under tropical conditions.

## Materials and methods

### Animals and treatment

The experimental animal procedures were following the animal research guidelines according to Kilkenny et al. (2010) and also the guidelines from Universiti Putra Malaysia. Trained staff and veterinarian were involved in blood samplings and monitoring the experimental animals to ensure minimal stress during the implementation of all experimental procedures.

The study was carried out at Advance Reproductive Biotechnology (ARB) farm, Kluang, Johor, Malaysia. A total of 127 cyclic Boer goats ranging in age from 4 to 6 years old, weighing 35 to 60 kg, and presenting a body condition score of 3.0 to 4.0 was used. All the experimental does were multiparous which previously gave birth more than once, and selected according to their health and fertility records. The goats were reared under semi-intensive management, grazing on *Brachiaria humidicola* and *Panicum maximum* pasture from 10 am until 4 pm and feeding on commercial pellets about 1% of body weight twice daily, before and after grazing. *Ad libitum* of clean water was provided every day. The does were randomly allocated to three treatment groups of estrus synchronization protocols.

**Figure 1:**
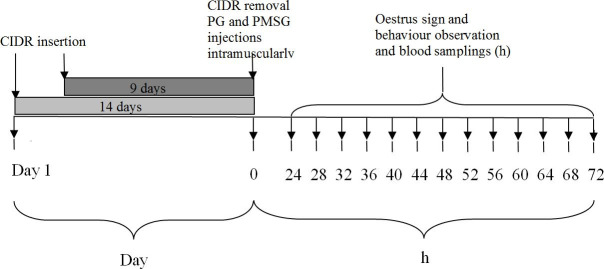
Diagram showing the time of the estrus synchronization, blood samplings, and estrus behaviour observation.

Control Internal Drug Release (CIDR) (Pfizer^®^, New Zealand) was inserted into the vagina of each goat for 14 days for estrus treatments PG14 (control) and PMSG14, and 9 days for estrus treatment PMSG9. Approximately 0.5 ml Estrumate^®^ (Cloprostenol 250 mg/mL) was injected intramuscularly to the does in all treatment groups during the withdrawal of the CIDR. Approximately 200 IU of Pregnant Mare Serum Gonadotropin (PMSG) FOLLIGON^®^ were injected intramuscularly to does in group PMSG14 and PMSG9 at CIDR withdrawal. Figure 1 shows the workflow of the experiment start from CIDR insertion to cessation of treatment and observations.

### Estrus signs observations

Estrus signs were observed 48 hours, post CIDR removal at 4 hours intervals (at 24, 28, 32, 36, 40, 44, 48, 52, 56, 60, 64, 68, and 72 h after CIDR removal). Each observation was conducted for at least 30 minutes. The estrus behaviour that has been recorded were mounting each other, standing to be mounted, tail wagging, bleating, and sniffing. The clinical signs of estrus are reddening of vulva, swollen vulva and discharge from vulva.

### Blood sampling and processing

Twelve does from each estrus synchronization protocol group were randomly selected for blood sampling. Approximately 4 ml of blood sample was collected from the jugular vein using a 21 G needle attached to a plain vacutainer tube (BD^®^ vacutainer, Plymouth, UK). Blood sampling was done before CIDR insertion, before CIDR removal, immediately after CIDR removal, and every 4 hours after CIDR removal until 48 hours. Blood was chilled overnight in the refrigerator (4°C) and then thawed at room temperature. Serum from the blood was separated by centrifugation at 500 *g* for 15 minutes. Then, the serum was transferred into a microcentrifuge tube and stored at -20°C before P4 and LH analysis.

### Serum analysis for P4 and LH

The serum samples collected before CIDR insertion, and withdrawal and 24 hours after withdrawal, were used to determine the P4 concentration. The serial serum samples collected at 24 hours after CIDR removal and thereafter at 4 hours intervals were used to determine LH concentration. The P4 and LH concentrations were determined using Enzyme-linked Immunosorbent Assay (ELISA) kit (Cusabio^®^, China). The concentration of the hormones was determined by optical density. The ELISA plate was read at 450 nm using a microtiter plate reader (Bio-Rad^®^ iMark Microplate reader). The coefficient of variation (CV) of intra-assay was 4.4% and inter-assay was and 11.7%. The minimum detectable levels were 0.2 ng/ml for P4 and 0.24 mIU/ml for LH.

### Statistical analysis

Data on estrus observations (does in estrus and specific estrus behaviour) were compared using the frequency procedure of SAS and Chi-square (χ^2^) test of independence. Data on LH and P4 hormones were analyzed using the GLM procedure of SAS software. Duncan Multiple Range Test (DMRT) was used to compare the mean. All statistical comparisons were set at a 0.05 level of significance.

### Compliance with ethical standards

The experimental animal procedures were following the animal research guidelines.

## Results

The duration of estrus interval between CIDR removal and the start of estrus, as well as the end of estrus (Table 1), showed no significant difference (P>0.05) among the treatment groups. However, treatment group PMSG9 showed better results when compared with treatment groups with 14 days of CIDR insertion. The interval from CIDR removal to the onset of estrus was shortest in treatment PMSG9 compared with the other two treatments. In addition, the does in treatment PMSG9 also had the longest duration of estrus expression than the other two treatment groups.

**Table 1:** Percentage of goats that responded to estrus synchronization treatments, the interval from CIDR removal to start of estrus, and end of estrus, and duration of estrus.

**Treatments**	**Estrus Synchronization Protocol**		
	**PMSG14**	**PG14**	**PMSG9**
N	41	42	41
% does responded (n)	97.56 (40)^a^	80.95 (34)^b^	100.0 (41)^a^
Time interval from CIDR removal and start of estrus (h)	29.2 ± 0.90	28.9 ± 0.99	27.7 ± 0.70
Time interval from CIDR removal and end Estrus(h)	54.1 ± 1.37	53.2 ± 2.19	57.3 ± 1.18
Duration of estrus (h)	24.9 ± 1.71	24.5 ± 2.32	29.6 ± 1.42

Means of a trait sharing common superscripts are not significantly different (P>0.05).

**Figure 2:**
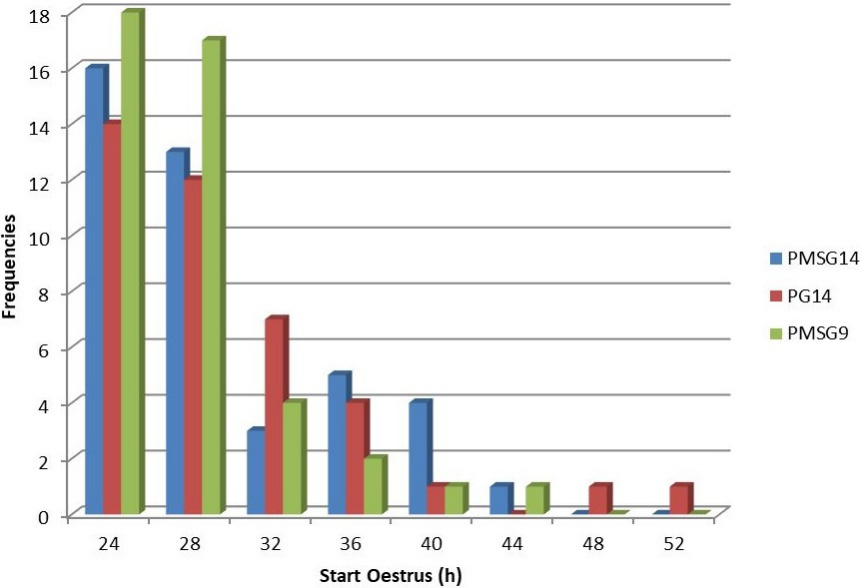
Distribution of time of does start their estrus signs after CIDR removal

Figure 2 shows the distribution of the start of estrus behaviour and signs from 24 h after CIDR removal. Most of the does display the estrus behaviour as early as 24 h and as late as 52 h after CIDR removal. About 81% of does from treatment PMSG9 started showing the estrus behaviour at 24^th^ h (n=18) and 28^th^ h (n=17) after CIDR removal. For treatments, PMSG14 and PG14 only about of the does 69% and 61% respectively, showed estrus behaviour by these times. The does start showing estrus behaviour for treatment groups with PMSG administration was the latest at 44 h. For the treatment group without the PMSG administration, some does start showing estrus behaviour only at 48-52 h after CIDR removal.

**Figure 3:**
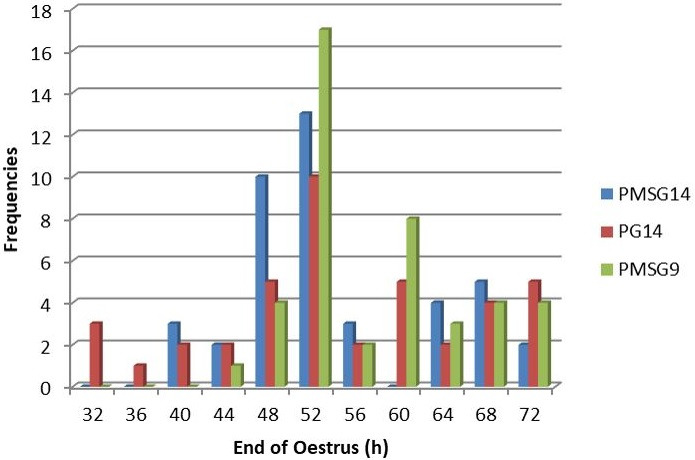
Distribution of time of does end their estrus signs after CIDR removal.

Figure 3 shows the distribution time of does end of the estrus signs. The range of end estrus was as early as 32 h after CIDR removal and as late as 72 h after CIDR removal. The highest frequencies of does end up their estrus sign were at 52 h after CIDR removal. In total, 43, 32, and 24% of does in treatment group PMSG9, PMSG14, PG14, respectively, end their estrus at 52 h after the CIDR removal. The rest of them does end their estrus behaviours and signs at several hours, which are about 10% of each treatment group end up at each hour of observation.

**Figure 4:**
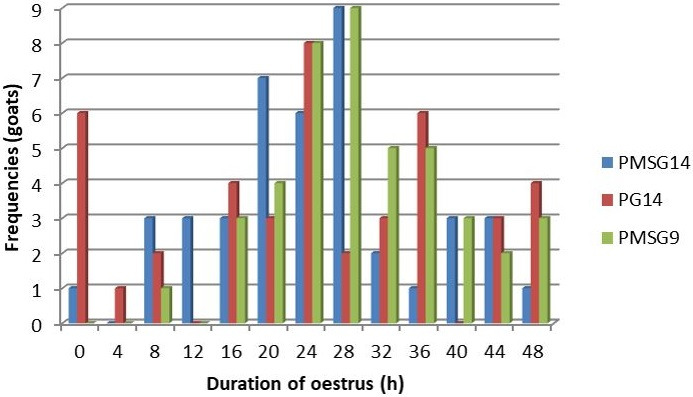
Distribution of estrus duration (h).

The duration of estrus was calculated from the time each doe starts showing estrus until the end of estrus. The duration of estrus ranged from 0 h (no sign of estrus) to 48 h (Figure 4). It shows, about 15% of does from the treatment group without the PMSG administration have not shown their estrus signs. A larger number of does in treatment groups with PMSG administration showed estrus duration for about 28 h. The frequency of estrus duration in group PG14 was inconsistent and ranged from 0 to 48 h. However, 20% of does in this group had 24 h estrus duration.

Table 2 gives the number of expressing estrus behaviour from 24 h to 72 h after CIDR removal under three estrus synchronization protocols. The most frequent behaviour exhibited by the does was tail wagging. Almost all of the does from the three treatment groups wagged their tails during estrus. Treatment groups with PMSG administration showed significantly (P<0.05) greater percentages of does express mounting, standing to be mounted, tail wagging, and sniffing behaviours compared to the treatment group without PMSG administration. Other estrus signs such as vulva discharge, swollen vulva, and vocalizing were not significantly different (P>0.05) among the treatment groups.

**Table 2:** Percentage (number) of does expressing different estrus signs.

	**Estrus Synchronization Protocol**		
	**PMSG14**	**PG14**	**PMSG9**
Behaviour expressed (%)			
Mounting	69.05 (29) ^a^	28.57 (12)^b^	65.12 (28) ^a^
Standing	69.05 (29) ^a^	28.57 (12)^b^	72.09 (31) ^a^
Tail wagging	95.24 (40) ^a^	83.33 (35)^b^	100 (43)^a^
Vulva Discharge	52.38 (22) ^a^	52.38 (22) ^a^	62.79 (27) ^a^
Swollen Vulva	71.43 (30) ^a^	66.67 (28) ^a^	81.40 (35) ^a^
Vocalizing	23.81 (10) ^a^	21.43 (9) ^a^	37.21 (16) ^a^
Sniffing	23.81 (10) ^a^	0.00 (0) ^b^	13.95 (6) ^a^

^a,b^Percentage value with different superscript within the same row are significantly different (P<0.05).

Table 3 shows the onset of estrus signs (mounting and standing to be mounted) behaviour after CIDR removal. Mounting behaviour showed no significant difference (P>0.05) between the treatment groups. As for standing to be mounted, this occurred significantly later (P<0.05) in the treatment without PMSG compared with groups with PMSG. Mounting and standing to be mounted behaviours were displayed at about the same time, about 32 to 44 h after CIDR removal.

**Table 3:** Mean of time between CIDR removal and receptivity behaviours (mounting and standing) behaviour (h ± SE).

**Treatment**	**PMSG14**	**PG14**	**PMSG9**
Mounting	36.14 ± 1.45^a^	35.38 ± 2.12^a^	36.00 ± 1.53^a^
n	29	13	25
Standing	36.66 ± 1.19^a^	42.00 ± 3.06^b^	37.39 ± 1.85^a^
n	29	12	31

^a,b^Mean values within the same row with different superscripts are significantly different at P<0.05.

There was no significant difference (P>0.05) in P4 concentration among all time points of P4 concentration analysis. Progesterone concentration before CIDR insertion ranged between 0.56 and 8.14 ng/ml. Twenty-four hours after the CIDR removal the concentration of the P4 hormone had generally declined except for the treatment group with 14 days CIDR insertion which were increases.

Table 4 shows the mean time of the occurrence of the LH peak from CIDR removal as well as the onset of estrus. The interval between CIDR removal and LH peak was significantly different (P<0.05) between treatment groups PG14 and PMSG9, but the interval from estrus onset was not significantly different (P>0.05).

**Table 4:** Intervals to LH peak occurrence, the interval from onset of estrus to LH peak (h±SE) and LH at peak (mIU/ml± SE).

**Treatment**	**PMSG14**	**PG14**	**PMSG9**
Interval from CIDR removal to LH peak (h) ± SE	39.2 ± 1.79^ab^	46.2 ± 4.17^a^	37.2 ± 1.89^b^
Interval from onset of estrus to LH peak (h) ± SE	11.2 ± 2.52^a^	16.0 ± 3.46^a^	9.60 ± 5.06^a^
Concentration of LH at peak (mIU/ml) ± SE	0.72 ± 0.08^a^	0.96 ± 0.12^a^	0.71 ± 0.10^a^

^a,b^Values within the same row with different superscripts are significantly different at P<0.05.

## Discussion

The percentage of does that responded to the estrus synchronization treatments were not significantly different (P>0.05) between the three treatment groups. However, treatment PG14 showed lower percentages of response compared to the group with PMSG administration regardless of the duration of insertion; 100% of does in the latter treatment exhibited estrus within 24 h to 72 h after CIDR removal and hormone treatment. This may be due to the absence of PMSG in the PG14 treatment group, PMSG plays a major role in stimulating follicular growth and subsequently leads to higher estrus response (Greyling and Van Niekerk, 1990a). This result was also in agreement with Oliveira et al. (2001) which were 100% of the Saanen does in their study expressed estrus when treated for 9 days with CIDR in combination with 100 IU of eCG and 0.05 mg of Cloprostenol at CIDR removal.

### PMSG administration helps the estrus synchronization

The time when the does start showing estrus was not significantly different (P>0.05) among the treatment groups. Most of the does show estrus as early as 24 h to 28 h after the CIDR removals. The treatment PMSG14 and PMSG9 showed significantly greater (P<0.05) frequencies 81% and 69%, respectively, as compared to treatment PG14 which was 61% of does from this group show estrus behaviour and signs by 24h and 28h of time. This may be also due to the PMSG administration. Often, PMSG is administered ranges from about 100 IU to 750 IU along with Cloprostenol (Wildeus, 2000; Oliveira et al., 2001; Abdalla et al., 2014). Whitley and Jackson (2004) reported that the inclusion of PMSG during estrus synchronization treatment may decrease the time interval between CIDR removal and the onset of estrus. The treatment group without PMSG showed a later occurrence of onset of estrus. Some of the onsets of estrus were only starting at 52 h after progestagen treatment. The results of the onset of estrus following progestagen withdrawal were not significantly different (P>0.05). The overall mean, 28.6±0.93 h was shorter than the result of Motlomelo et al. (2002), Hashemi and Safdarian (2018), and Romano (2004) who reported intervals of 30.1±5.5 h, 36 h, and 40.2±10.5 h, respectively. The differences in estrus duration reported by the different research probably contributed by several factors such as breed, nutrition, and environment (Ahmed et al., 1998; Motlomelo et al., 2002; Lehloenya et al., 2005).

The duration of estrus obtained from this study was shorter (24.5 ± 2.32 to 29.6 ± 1.42 h) compared to findings from Motlomelo et al. (2002) 35.2 ± 0.7 h. Lehloenya et al. (2005) reported the duration of induced estrus in Boer goats was (37.0 h) longer than the present study. Compared to other types of pessaries such as FGA (fluorogestone acetate) sponge with PMSG, the duration of estrus can go up to 52±7.8 h. The duration of estrus was longer than 48 h maybe because the observation time started only at 24 h and ended at 72 h after CIDR removal. PMSG helped in tighten the duration of estrus, hence, the estrus could be limit until 72 h after CIDR removal (Wheaton et al., 1993). As found in a study by Oliveira et al. (2001), the estrus occurrence was only about 24 h when treated with PMSG which may have promoted the occurrence of a pre-ovulation LH peak and induced premature ovulation. The onset and duration of estrus may also be influenced by breeds, seasons, and nutrition (Dogan et al., 2008).

### Estrus behaviour observations

There were three stages of animal showing estrus behaviour; attractivity, proceptivity, and receptivity. The attractivity and proceptivity behaviour are always expressed together for example sniffing the genitalia area, tail wagging, and mounting behaviour (Beach, 1976). These behaviours are also known as initiation before copulation occurs. During this time, animals usually seek males and try to attract them. The inclusion of PMSG in the estrus synchronization protocol may help in raise the estrogen in blood serum and subsequently facilitate the expression of these estrus behaviour (Imwalle and Katz, 2004). Mounting and standing are the gestures that confirm the animal is in estrus and ready for copulation (Katz and McDonald, 1992). The inclusion of PMSG may also contribute to the high percentage of receptivity behaviour and will result in a higher percentage of fertility. The occurrence of receptivity behaviour (standing to be mounted) is always supported with mounting behaviour. These behaviours are also related to the occurrence of the pre-ovulatory LH surge. Fabre-Nys and Martin (1991) reported that the pre-ovulatory LH surge falls within the time of proceptive and receptive behaviour in does. These behaviours were analyzed and it showed no significant differences between each treatment group. The range time of mounting and standing behaviour which were between 32 h and 44 h) might influence the LH peak to fall within this range of time.

Comparing the time of CIDR in the vagina, 14 or 9 days did not give any significant difference in does express estrus behaviour, especially the receptivity behaviour. It is because the duration of progesterone contained device like CIDR in the vagina may not differ in the effects. After all, it was the same route although different duration. Another study had been reported, that the time of P4 hormone administration or duration did not significantly affect the receptivity behaviour in goats, but the latter was influenced by seasons and route of administration (Billings and Katz, 1999).

### Hormonal pattern after the drug administrations

The P4 hormone concentration showed no differences among sampling time (before CIDR insertion and 24 h after CIDR removal). It was relatively low for all treatment groups after CIDR removal with a range of 0.302 to 7.705 ng/ml. These data were in agreement with (Greyling and Van der Nest, 2000) who reported that the ranges of the P4 hormone after intravaginal device being removed as 0.01 to 9.05 ng/ml. In contrast, the study from (Greyling and Van Niekerk, 1990b) had reported the concentrations of P4 hormone during the onset of estrus ranged between 0.35 and 0.6 ng/ml in Boer goat. Probably the standard concentrations being provided by the manufacturer of the ELISA kit which has ranges 0 ng/ml to 50 ng/ml of P4 hormone concentration might have affected the variation and the sensitivity of the antibody in the kit. Greyling and Van Niekerk (1990b) had used, modified method from Van der Westhuysen (1979) which was used ranges 0 – 10 ng/ml of P4 hormone concentration. During the time of estrus, the P4 hormone concentration should remain low to trigger the LH surge and subsequently ovulation occurred. The P4 hormone concentration will decrease as the time approaching ovulation due to the administration of Cloprostenol (Fonseca et al., 2008).

The time interval between CIDR removal and LH peak occurrence was varied among the treatment groups. It may be due to different types of hormones used for synchronization. The administration of PMSG fastens the occurrence of the preovulatory LH peak (Pierson et al., 2001). The PMSG contained glycoprotein hormone that would possess the FSH as well as LH. However, the time interval between the onset of estrus and LH peak occurrence showed no differences (P>0.05) between each treatment group. The ranges of time interval onset of estrus to the occurrence of the LH peak were 4 h to 20 h and it was in agreement with Baril and Vallet (1990) who reported the interval about 4 h to 16 h and 4h to 28 h for breeding and non-breeding season, respectively.

The LH peak concentrations of this study were contradicted with Gaafar et al. (2005) that had been reported the concentration was about 33.6 mIU/ml and 59.3 mIU/ml. While this study only recorded a maximum concentration of about 1.06 mIU/ml. It was different probably because the method of hormone detection was different. Gaafar et al. (2005) was using Radioimmunoassay (RIA) kit compared to this study using different methods (ELISA) kit. On the other hand, it also may be due to the sensitivity of the ELISA kit (CUSABIO^®^) were not enough to detect the LH surge in the blood serum. Martínez-Álvarez et al. (2007) reported, the variation of the time interval between progestagen treatment and the onset of estrus, LH peak, and ovulation was affected by the type of progestagen, route of administration and dose of drugs being used, and duration of treatment. The ovulation time is unknown and is only predicted by the occurrence of the LH peak. However, the ranges of time for the occurrence of the LH peak were still relevant to other studies mentioned above.

## Conclusion

In a nutshell, the suitable protocol estrus synchronization that farmers may choose is protocol 14 or 9 days of CIDR in conjunction with PG and PMSG because it gives a better success rate in Boer does showing estrus behaviour compared to protocol group without PMSG. The administration of PMSG may help in tighten estrus and improved fertility Boer goat does.
